# Genetic association study of circadian genes with seasonal pattern in bipolar disorders

**DOI:** 10.1038/srep10232

**Published:** 2015-05-19

**Authors:** Pierre Alexis Geoffroy, Mohamed Lajnef, Frank Bellivier, Stéphane Jamain, Sébastien Gard, Jean-Pierre Kahn, Chantal Henry, Marion Leboyer, Bruno Etain

**Affiliations:** 1 Inserm, U1144, Paris, F-75006, France; 2 AP-HP, GH Saint-Louis - Lariboisière - Fernand Widal, Pôle Neurosciences, 75475 Paris Cedex 10, France; 3 Université Paris Descartes, UMR-S 1144, Paris, F-75006, France; 4 Fondation FondaMental, Créteil, 94000, France; 5 INSERM, U955, Psychiatrie génétique, Créteil, 94000, France; 6 AP-HP, Hôpitaux Universitaires Albert Chenevier-Henri Mondor , DHU PePSY, Pôle de Psychiatrie, Créteil, 94000, France; 7 Université Paris Est, Faculté de médecine, Créteil, 94000, France; 8 Hôpital Charles Perrens, Centre Expert Trouble Bipolaire, Service de psychiatrie adulte, Pôle 3-4-7, Bordeaux, 33000, France; 9 Service de Psychiatrie et Psychologie Clinique, CHU de Nancy, Hôpitaux de Brabois, Vandoeuvre Les Nancy, 54500, France

## Abstract

About one fourth of patients with bipolar disorders (BD) have depressive episodes with a seasonal pattern (SP) coupled to a more severe disease. However, the underlying genetic influence on a SP in BD remains to be identified. We studied 269 BD Caucasian patients, with and without SP, recruited from university-affiliated psychiatric departments in France and performed a genetic single-marker analysis followed by a gene-based analysis on 349 single nucleotide polymorphisms (SNPs) spanning 21 circadian genes and 3 melatonin pathway genes. A SP in BD was nominally associated with 14 SNPs identified in 6 circadian genes: *NPAS2*, *CRY2*, *ARNTL*, *ARNTL2*, *RORA* and *RORB*. After correcting for multiple testing, using a false discovery rate approach, the associations remained significant for 5 SNPs in *NPAS2* (chromosome 2:100793045–100989719): rs6738097 (p_c_ = 0.006), rs12622050 (p_c_ = 0.006), rs2305159 (p_c_ = 0.01), rs1542179 (p_c_ = 0.01), and rs1562313 (p_c_ = 0.02). The gene-based analysis of the 349 SNPs showed that rs6738097 (*NPAS2*) and rs1554338 (*CRY2*) were significantly associated with the SP phenotype (respective Empirical p-values of 0.0003 and 0.005). The associations remained significant for rs6738097 (NPAS2) after Bonferroni correction. The epistasis analysis between rs6738097 (*NPAS2*) and rs1554338 (*CRY2*) suggested an additive effect. Genetic variations in *NPAS2* might be a biomarker for a seasonal pattern in BD.

Bipolar disorders (BD) are severe and chronic mental disorders affecting 1–4% of the population worldwide^1^. BD are defined by episodes of major depression and hypomanic or manic phases, with intermittent periods of remission. They are ranked as one of the most burdensome diseases globally due to their peak age at onset in adolescence and early adulthood, consequently leading to poor functioning and diminished quality of life throughout adulthood^2^,^3^,^4^.

BD are associated with major disruptions in circadian rhythms, including abnormal sleep/wake cycles, as well as alterations in biochemical, appetite, seasonal, and social rhythms^5^,^6^. Circadian rhythms are generated and synchronized by the endogenous cellular clock located in the suprachiasmatic nucleus of the hypothalamus in all mammals^7^,^8^. Alterations in this endogenous machinery regulating circadian oscillations may be involved in the susceptibility to mood disorders including BD, unipolar major depressive disorder (MDD) and seasonal affective disorder (SAD)^8^. Pineal gland melatonin secretion, another key regulator of circadian rhythms and sleep patterns pertinent to the circadian molecular machinery, has also been shown to be altered in BD^9^,^10^.

Furthermore, (hypo)manic and depressive phases in BD patients can follow a seasonal pattern (SP). This SP can be observed from several perspectives when examining admission rates for acute episodes, seasonal recurrence of mood episodes and fluctuations in symptoms^11^. These infradian fluctuations are very frequent in BD for depressive episodes (25%) as well as manic episodes (15%), according to DSM criteria^11^. In comparison to MDD, the one in four prevalence of seasonal depression in BD exceeds the 10-20% prevalence found in MDD outpatients^12^, with BD seasonal depression showing an odds ratio of around 4 in comparison to primary care populations^13^. We recently confirmed that the frequency of SP according to DSM-IV-TR criteria is around 25% in a population of BD outpatients^14^. We also showed that patients with SP exhibited a more severe disease characterized by rapid cycling, BD II subtype, comorbid eating disorders, more mood episodes (especially depressions) and younger age of onset^14^. However, the underlying genetic factors driving SP in BD remains to be identified, with circadian genes being potential candidates. It is important to note that SP is thought to be heritable and therefore driven, at least in part, by genetic variants^15^,^16^,^17^. Among candidate genes, ‘core clock genes’ and melatoninergic related genes are putative candidates. These genes have been mainly investigated for their role in the genetic susceptibility to BD per se, but not for their influence on SP. Indeed, clock and melatonin pathways closely interact in chronobiological mechanisms related to photoperiod regulation, being crucial factors in the adaptation to seasonal variations, as well as in the synchronization of not only circadian but also infradian rhythms^18^. BD patients are supersensitive to melatonin suppression by light^19^,^20^,^21^, which has been proposed as a “trait” BD biomarker, given its independence of patient mood state, strongly heritability and increasing prevalence with increased familial genetic load^21^,^22^. As such, melatonin abnormalities could be relevant to alterations in the rhythms of the subjective day as well as the time of year, being therefore implicated in seasonal sensitivity to mood fluctuations.

To date, only one study has been published that directly investigated the genetic influence on SP in BD patients^23^. These authors, using a genome-wide association study (GWAS) approach, investigated seasonal manic episodes, identifying several relevant genomic regions, with the most significant association being obtained within an intron of the nuclear factor-1A (*NF1A)* gene, which is involved in cellular transcription and DNA replication^23^. No study on seasonal depressive episodes in BD has been performed to date. However, in populations meeting the DSM-IV criteria for MDD or BD with a winter seasonnality, two studies report that the development of SAD is associated with variants of circadian and melatoninergic genes, suggesting associations with an amino acid substitution in *NPAS2* (471 Leu/Ser)^24^, as well as with a combination of variations in 3 circadian genes *PER2, ARNTL (Bmal1), and NPAS2*^25^. In regard to the melatonin pathway, an association for SP in BD or MDD patients was found for the X-linked *GPR50* gene (coding for an orphan member of the G protein-coupled melatonin receptor subfamily), although only in women^26^. Such lines of evidence led us to hypothesize that circadian genes contribute to the susceptibility to a depressive SP in BD.

## Aim of the study

The aim of this study was to investigate the association between 24 circadian genes with SP in BD.

## Materials and Methods

### Sample

French Caucasian individuals who met DSM-IV criteria^27^ for BD I or BD II were recruited from three university-affiliated psychiatric departments in France (Paris, Bordeaux, Nancy). This study was approved by the medical ethics committee of the French « Comité de Protection des Personnes (CPP) » (IDRCB2008_AO1465_50 VI - Pitié Salpêtrière 118-08) and carried out in accordance with the approved guidelines. With institutional review board ethics approval, participants who gave written informed consent were assessed by a psychiatrist or psychologist trained in the use of the French version of the Diagnostic Interview for Genetic Studies (DIGS). The DIGS is a structured interview schedule that provides a retrospective lifetime diagnosis of BD, treatments and other axis I disorders meeting DSM IV criteria^28^. Both familial and sporadic cases were included in the study. Patients had to be in symptomatic remission for at least three months and with scores on the Montgomery-Åsberg depression Rating Scale (MADRS) and the Young Mania Rating Scale (YMRS) lower than 5 at inclusion.

### Phenotypes

Seasonal pattern (SP) was defined according to DSM-IV TR criteria^27^

1. Regular temporal relationship between the onset of major depressive episodes and a particular time of the year (unrelated to obvious season-related psychosocial stressors),
2. Full remissions (or a change from depression to mania or hypomania) also occur at a characteristic time of the year,
3. Two major depressive episodes meeting criteria 1 and 2 in the past two years and no non-seasonal episodes in the same period,
4. Seasonal major depressive episodes substantially outnumber the non-seasonal episodes over the individual’s lifetime.

### Genotypic data and statistical analyses

Genomic DNA samples were extracted from peripheral blood leukocytes or B-lymphoblastoid cell lines by standard procedures. Genotyping was performed at the Centre National de Génotypage (CNG, CEA, France) using HumanHap550 or 610-Quad Beadchips (Illumina Inc., San Diego, CA, U.S.A.) for bipolar patients, and HumanHap300 Beadchips (Illumina Inc.) for controls^29^. Data for 349 single nucleotide polymorphisms (SNPs) spanning 21 circadian genes were extracted (*CLOCK*, *NPAS2*, *ARNTL1*, *ARNTL2*, *PER1*, *PER2*, *PER3*, *CRY1*, *CRY2*, *TIMELESS*, *NR1D1*, *RORA*, *RORB*, *RORC*, *CSNK1δ*, *CSNK1ε*, *GSK3β*, *DBP*, *BHLHB2*, *BHLHB3*, *PPARGC1A*) as previously described^10^ to which 3 melatonin pathway genes (melatonin receptors 1a and 1b (*MTNR1a* and *MTNR1b*), and arylalkylamine N-acetyltransferase (*AANAT*) have been added^5^,^9^. Table S1 summarizes the localization of the studied genes. Genes were selected, based on their involvement in the molecular mechanisms that regulate circadian rhythms and mediate the downstream effects of melatonin. All available SNPs within each gene and within 10 kilobase pairs (Kbp) upstream and downstream from the coding sequence (extracted from the RefSeq Database (National Center for Biotechnology Information) were used to explore exonic and intronic regions, as well as cis-regulatory regions.

SNPs were included in the association analyses if they fulfilled all of following quality criteria, assessed using PLINK software^30^:

1. Minor allele frequencies (MAF) greater than or equal to 5%.
2. Genotyping call rate for at least 97% of SNPs.
3. The call rate averaged 99.8%.
4. A SNP heterozygosis between m-3sd and m + 3sd.

### Genetic single-marker analysis

For each SNP, we performed an association study between the SNP and SP (presence versus absence) by using Chi square tests to compare allelic distributions between SP and non-SP. To take into account the multiple tests while controlling for the risk of false discovery, we used a false-discovery-rate (FDR) approach^31^. All association analysis were performed using PLINK v1.07^30^ and R version 3.0.1 (2013-05-16) software.

### Gene based analysis

Compared to a single-marker analysis, the gene-based analysis has several methodological advantages. Single-marker tests may indeed be inefficient if each single marker carries a small to moderate amount of association information about the trait or if there is allelic heterogeneity^32^. However, false positive results might emerge for a single SNP analysis. The advantages of gene-based tests are that they: 1) combine information on markers within a gene; and 2) account for differences in gene size^33^.

Each gene was analyzed using the set-based test in PLINK v1.07 30. Briefly, for each gene, the program: 1) determines which SNPs are in linkage disequilibrium (LD) (above a certain threshold, fixed in this study at r^2^ = 0.2); 2) performs a single SNP association analysis (Fisher’s test); and (3) determines the associated SNP having the highest statistical significance and then ranks the other SNPs by decreasing statistical significance, after removing SNPs in LD with previously selected SNPs^33^. We fixed the maximum number of selected SNPs at 3 (the smallest number of SNPs per gene among all the genes studied). The statistic of each gene was then calculated as the mean of these single SNP statistics. Empirical p-values for each gene were estimated by using 10,000 permutations of the dataset and consequently were corrected for multiple testing within the gene and accounted for LD between the SNPs of the gene. In this way, a Bonferroni correction was applied to these p-values according to the number of genes tested (n = 24) corresponding to a significance threshold of 0.002 (alpha = 0.05/24).

### Epistasis analysis

The epistasis analysis was then performed to test SNPxSNP interaction on SP, by testing the best SNPs from genes identified by the gene-based analysis. This test for interaction uses logistic regression and was performed using PLINK v1.07 30.

## Results

Data for 349 SNPs spanning 24 circadian and melatonin genes were examined in a sample of 269 BD patients. Seventy patients presented with SP (26%) and 199 without SP (74%).

Patients with BD and SP demonstrated more mood recurrences (p = 0.0008), an earlier age at onset (p = 0.03) and were more associated with a BD II subtype (p = 0.04), than those without SP. In contrast, no differences were observed between groups regarding gender, age, duration of BD and education levels (Table 1).

Single-marker analysis identified 14 nominal associations (uncorrected p < 0.05) between SP and SNPs located in 6 circadian genes: *NPAS2* (7 SNPs), *CRY2* (1 SNP), *ARNTL* (1 SNP), *ARNTL2* (1 SNP), *RORA* (2 SNPs) and *RORB* (2 SNPs) (Table 2).

After correcting for multiple testing using the FDR approach, the associations remained significant across NPAS2 (chromosome 2:100793045-100989719) for 5 SNPs: rs6738097 (p_c_ = 6.10^−3^), rs12622050 (p_c_ = 6.10^−3^), rs2305159 (p_c_ = 0.01), rs1542179 (p_c_ = 0.01), and rs1562313 (p_c_ = 0.02) (Table 3). These 5 SNPs are localized in the *NPAS2* introns.

Among the total 349 SNPs tested by gene-based approach, rs6738097 (*NPAS2*) and rs1554338 (*CRY2*) were significantly associated with the SP phenotype (Empirical p-value = 0.0003 and Empirical p-value = 0.005 respectively). The associations remained significant only for rs6738097 (*NPAS2*) after Bonferroni correction for the 24 genes tested (p < 0.002) (Table 3).

Following the gene-based approach analysis, we tested the interaction between the two best SNPs identified, namely rs6738097 (*NPAS2*) and rs1554338 (*CRY2*). This epistasis analysis did not appear statistically significant (OR = 3.18; Chi-square statistic = 2.16; p = 0.14) and suggested an additive effect rather than an interaction.

## Discussion

This genetic association study, examining clock and melatonin gene variants, found several nominal associations between SP and SNPs in 6 circadian genes: *NPAS2, CRY2, ARNTL, ARNTL2, RORA* and *RORB*. The association with the *NPAS2* gene could be considered as robust since our genetic analyses showed significant associations that survived corrections for multiple testing. An additive effect on SP in BD is suggested for rs6738097 (*NPAS2*) and rs1554338 (*CRY2*).

Whereas this study is the first one to associate *NPAS2* and SP in a BD population, previous results have highlighted a putative role of the circadian gene *NPAS2* in BD susceptibility more generally, and probably more specifically to BD with SP. Indeed, 3 studies have previously found associations of *NPAS2* gene with BD^34^,^35^,^36^, and a winter SP with either MDD or BD populations being associated with: i) an amino acid substitution in *NPAS2* (471 Leu/Ser)^24^; and ii) a combination of variations in 3 circadian genes that include *NPAS2* (*PER2, ARNTL, and NPAS2)*^25^. An additional study found an association of *NPAS2* with MDD^36^.

From a molecular point of view, the NPAS2 protein encoded by this circadian gene is a member of the basic helix-loop-helix (bHLH)-PAS family of transcription factors^37^. Briefly, the mammalian clock is regulated at the cellular level by a transcriptional/translational feedback loop. BMAL1/NPAS2 heterodimers activate the expression of the period (*PER*) and cryptochrome (*CRY*) genes acting as transcription factors directed to the *PER* and *CRY* promoters via E-box elements. PER and CRY proteins form heterodimers and suppress the activity of the BMAL1/NPAS2 completing the feedback loop. The circadian expression of *NPAS2* is influenced by retinoic acid receptor-related orphan receptor-alpha (RORα) and REV-ERBα, two nuclear receptors that target a ROR-response element in the promoter of the *NPAS2* gene^38^. Although having a crucial role in regulating circadian rhythms, the precise role of *NPAS2* in sensitivity to photoperiod variations and the regulation of infradian rhythms remain to be clarified in animal models and humans.

Nevertheless, the *NPAS2* gene has already been associated with later sleep and wake onset time in an elderly patients with osteoporosis^39^, but also with poorer adaptation to shift work in nurses that presented with more alcohol/caffeine consumption, sleepiness, and disturbances of sleep phase, inertia and duration^40^. A recent GWAS meta-analysis in drosophilae also associated *NPAS2* with sleep duration^41^. Our data here contributes to the idea of a role of the *NPAS2* gene in BD susceptibility. Interestingly, the *NPAS2* gene has also been associated with other disorders or physiological functions that involve abnormalities n the regulation of circadian rhythms, such as prostate cancer^42^, breast cancer^43^, fertility and seasonality^44^, responses to restricted feeding^45^, and chronic fatigue syndrome^46^. Furthermore, beyond its implication in chronobiology, *NPAS2* gene has also been suggested a transcriptional regulator and a putative tumor suppressor in breast cancer^47^.

This study also has some limitations. Firstly, the sample was only of moderate size, although this size could be considered as adequate for a hypothesis-driven association study. However, a larger sample would have increased the power for detecting small effects and a larger study group population should probably be recommended for future studies of SP in BD. Secondly, we explored a large number of SNPs that could inflate the risk of type I errors; however, we used stringent correction for multiple testing to limit this. Thirdly, the SP phenotype was restricted to DSM-IV definition for depressive episodes. The consideration of the DSM-5 specifier criteria for SP (noting mood episodes of any polarity) will allow for the examination of SP in BD (hypo)manic episodes in future studies^48^. Finally, the acetylserotonin O-methyltransferase (*ASMT*) gene, encoding one of the enzymes involved in melatonin biosynthesis, is an important putative susceptibility gene for BD^9^,^49^ but was not examined in our study because of its unavailability in our DNA chip. However, in a post-hoc analysis, we examined four SNPs (rs4446909, rs5989681, rs56690322, rs6644635) in the *ASMT* promoter, but did not find any significant associations (data not shown).

To conclude, very little is known concerning the role of circadian genes in the common susceptibility to infradian rhythmicity abnormality in BD. This first genetic association study in a BD population, thanks to both genetic single-marker and gene based analyses, demonstrates that genetic variations in *NPAS2* might be a relevant marker for the susceptibility to BD with a SP. Others circadian genes may deserve future attention because of nominal associations with SP in our sample, such as *CRY2*, *ARNTL*, *ARNTL2*, *RORA* and *RORB.* However, our results regarding *NPAS2* may provide promise in improving clinical practice in the diagnosis and treatment of BD. Indeed, for breast cancer patients, a high level of NPAS2 in cancer cells has been strongly associated with improved disease free survival time as well as overall survival time^43^. Whereas prognostic significance of the circadian gene *NPAS2* in breast cancer is suggested, its application and relevance in BD requires specific testing in future studies, which, if confirmed, may serve as a predictor of prognosis. Moreover, the exact role that *NPAS2* variants play in mood disorders vulnerability will need to be further examined, and may pave the way for personalized circadian therapy in the management of mental health. More generally, these results open news avenues for future researches in variants of circadian genes that may help for the development of a next generation of drugs that target sleep and circadian rhythm pathways. Nevertheless, to be added to the pharmacological arsenal for BD treatment, these researches should also clarify the associations between sleep and circadian rhythm disturbances and candidate variants of circadian genes in order to identify critical targets within the circadian pathway.

## Author Contributions

P.A.G. & B.E. designed the study. P.A.G., M.L.a, F.B., S.J., S.G., J.P.K., C.H., M.L.e & B.E. collected the data. P.A.G., M.L.a & B.E. made the analyses. P.A.G., M.La, F.B., S.J., S.G., J.P.K., C.H., M.Le & B.E. participated in the interpretation of results, the manuscript redaction and approved its final version.

## Additional Information

**How to cite this article**: Geoffroy, P. A. *et al*. Genetic association study of circadian genes with seasonal pattern in bipolar disorders. *Sci. Rep.* **5**, 10232; doi: 10.1038/srep10232 (2015).

## Supplementary Material

Supplementary Table 1

## Figures and Tables

**Table 1:** **Socio-demographic and clinical characteristics of patients with (SP+) and without seasonal pattern (SP−).**

**Variables**	**N (269)**	**SP+(n=70, 26%)**	**SP− (n=199, 74%)**	**Z/Chi**^2^	***P value***
		**mean (SD) or n (%)**^a^			
**Gender**					
Female	269	45 (64.3)	129 (64.8)	0.01	0.93
Male		25 (35.2)	70 (35.2)		
**Age**	269	43.80 (13.1)	44.90 (13.2)	0.75	0.42
**Duration**	265	21.13 (12.3)	18.51 (11)	1.43	0.15
**Total number of depressive episodes**	269	9.83 (8)	5.14 (4.5)	5.40	**<0.0001**
**Total number of manic episodes**	264	3.10 (5.6)	1.93 (2.8)	0.43	0.67
**Age at onset**	267	22.69 (8.5)	26.30 (11.3)	2.13	**0.03**
**Bipolar disorder subtype**					
I	269	42 (60)	145 (72.9)	4.04	**0.04**
II		28 (40)	54 (27.1)		
**Education**					
Moderate level (<BAC +2)	269	42 (60)	115 (57.8)	0.10	0.74
High level (>BAC +2)		28 (40)	84 (42.2)		

BAC: bachelor degree

**Table 2:** **Nominal associations of the genetic single-marker analysis of circadian genes and Seasonal Pattern (SP) in Bipolar Disorders.**

**Chromosome**	**Gene**	**SNP**	**BP**	**A1**	**F SP**	**F no SP**	**A2**	**CHISQ**	**OR**	**L95**	**U95**	***p***
2	*NPAS2*	rs6738097	100944974	C	0.24	0.10	T	17.69	2.87	1,73	4,76	2.597e-05
2	*NPAS2*	rs12622050	100945886	A	0.32	0.15	G	17.18	2.51	1,61	3,93	3.401e-05
2	*NPAS2*	rs2305159	100957875	C	0.36	0.20	A	14.99	2.27	1,49	3,47	0.00010
2	*NPAS2*	rs1542179	100961667	G	0.36	0.20	A	14.51	2.25	1,47	3,45	0.00013
2	*NPAS2*	rs1562313	100953887	T	0.28	0.14	C	12.84	2.28	1,44	3,61	0.00033
11	*CRY2*	rs1554338	45863406	G	0.13	0.05	A	10.35	2.81	1,46	5,41	0.0012
11	*ARNTL*	rs3816358	13348048	A	0.05	0.13	C	6.90	0.35	0,15	0,79	0.0086
2	*NPAS2*	rs2278722	100986568	G	0.20	0.11	A	5.79	1.86	1,11	3,12	0.016
12	*ARNTL2*	rs2968756	27391721	G	0.17	0.097	A	5.69	1.93	1,11	3,36	0.017
2	*NPAS2*	rs11894671	100896250	T	0.17	0.26	G	4.61	0.58	0,36	0,96	0.031
15	*RORA*	rs782947	59126553	C	0.18	0.11	T	4.39	1.74	1,03	2,95	0.036
9	*RORB*	rs1885967	76488246	G	0.20	0.12	A	4.26	1.70	1,02	2,83	0.038
9	*RORB*	rs1327836	76482109	G	0.20	0.13	T	4.05	1.66	1,01	2,74	0.044
15	*RORA*	rs7179259	58945349	C	0.47	0.37	T	3.99	1.48	1,00	2,2	0.045

A1: Minor allele name (based on whole sample)

BP: Physical position (base-pair)

F SP: Minor allele frequency in subjects with SP

F; no SP: Minor allele frequency in subjects without SP

OR: Odds ratio

SNP: single nucleotide polymorphism

**Table 3:** Significant associations of the genetic single-marker analysis and gene based analysis of circadian genes and Seasonal Pattern (SP) in Bipolar Disorders.
