# Supplementary material for: Genetic association study of circadian genes with seasonal pattern in bipolar disorders
Source: Sci Rep. 2015 May 19;5:10232. doi: 10.1038/srep10232 (PMC4437291; doi:10.1038/srep10232)
Supplement: Supplementary Table 1 [file srep10232-s1.doc]

**GENETIC ASSOCIATION STUDY OF CIRCADIAN GENES
WITH SEASONAL PATTERN IN BIPOLAR DISORDERS**

***Runnning title:*** Circadian genes and Seasonal Bipolar Disorders.

Pierre Alexis **GEOFFROY**^1,2,3,4*^, Mohamed **LAJNEF**^4,5,6^, Frank **BELLIVIER**^1,2,3,4^,
Stéphane **JAMAIN**^4,5,7^, Sébastien **GARD**^4,8^, Jean-Pierre **KAHN**^4,9^,

Chantal **HENRY**^4,5,6,7^, Marion **LEBOYER**^4,5,6,7^, Bruno **ETAIN**^4,5,6,7^.

1) Inserm, U1144, Paris, F-75006, France;

2) AP-HP, GH Saint-Louis - Lariboisière - Fernand Widal, Pôle Neurosciences, 75475 Paris Cedex 10, France;

3) Université Paris Descartes, UMR-S 1144, Paris, F-75006, France & Université Paris Diderot, UMR-S 1144, Paris, F-75013, France ;

4) Fondation FondaMental, Créteil, 94000, France;

5) INSERM, U955, Psychiatrie génétique, Créteil, 94000, France;

6) AP-HP, Hôpitaux Universitaires Albert Chenevier-Henri Mondor , DHU PePSY, Pôle de Psychiatrie, Créteil, 94000, France;

7) Université Paris Est, Faculté de médecine, Créteil, 94000, France ;

8) Hôpital Charles Perrens, Centre Expert Trouble Bipolaire, Service de psychiatrie adulte, Pôle 3-4-7, Bordeaux, 33000, France;

9) Service de Psychiatrie et Psychologie Clinique, CHU de Nancy, Hôpitaux de Brabois, Vandoeuvre Les Nancy, 54500, France.

****Correspondence***

**Pierre Alexis Geoffroy, MD, MSc**

Service de Psychiatrie Adulte (Pr Bellivier)
Hôpital Fernand Widal
200, rue du Faubourg Saint-Denis
75475 Paris Cedex 10, France.
Tel: + 33 1 40 05 48 69 - Fax: + 33 1 40 05 49 33
E-mail: [pierre.a.geoffroy@gmail.com](mailto:pierre.a.geoffroy@gmail.com)

***Original research***

***Manuscript:*** 2718 words (***Abstract:*** 200/200)

***3 Tables & 1 supplemental Table***

Table S1. Localisation of the 21 circadian genes and 3 melatonin genes analysed for this study

| **Chromosome** | **Position -10kb** | **Position +10kb** | **Gene Name** |
| --- | --- | --- | --- |
| ***Circadian genes*** | | | |
| 1 | 7757301 | 7837824 | *PER3* |
| 1 | 150035170 | 150080972 | *RORC* |
| 2 | 100793045 | 100989719 | *NPAS2* |
| 2 | 238807418 | 238871946 | *PER2* |
| 3 | 4986208 | 5011863 | *BHLHE40* |
| 3 | 121018236 | 121305203 | *GSK3B* |
| 4 | 23392742 | 23510798 | *PPARGC1A* |
| 4 | 55983417 | 56117754 | *CLOCK* |
| 9 | 76292072 | 76501937 | *RORB* |
| 11 | 13245901 | 13375388 | *ARNTL* |
| 11 | 45815533 | 45871375 | *CRY2* |
| 12 | 26154226 | 26179113 | *BHLHE41* |
| 12 | 27367255 | 27474733 | *ARNTL2* |
| 12 | 55087174 | 55139467 | *TIMELESS* |
| 12 | 105899272 | 106021728 | *CRY1* |
| 15 | 58575776 | 59318794 | *RORA* |
| 17 | 7974515 | 8006478 | *PER1* |
| 17 | 35492567 | 35520499 | *NR1D1* |
| 17 | 77785529 | 77834862 | *CSNK1D* |
| 19 | 53815629 | 53842451 | *DBP* |
| 22 | 37024642 | 37054035 | *CSNK1E* |
| ***Melatonin genes*** | | | |
| 4 | 187681803 | 187723531 | *MTNR1A* |
| 11 | 92332437 | 92365596 | *MTNR1B* |
| 17 | 71965246 | 71987794 | *AANAT* |
